# Factors associated with paradoxical immune response to antiretroviral therapy in HIV infected patients: a case control study

**DOI:** 10.1186/1471-2334-11-306

**Published:** 2011-11-02

**Authors:** Janaina AS Casotti, Luciana N Passos, Fabiano JP Oliveira, Crispim Cerutti

**Affiliations:** 1 Infectious Diseases Outpatient Clinic of the Hospital Universitário Cassiano Antonio Moraes of Universidade Federal do Espírito Santo, Vitória, Espírito Santo State, Brazil; 2 Secretary of Health of the Municipality of Vitória, Vitória, Espírito Santo State, Brazil; 3 Tropical Medicine Unit and Program of Post Graduation in Collective Health of the Universidade Federal do Espírito Santo, Vitória, Espírito Santo State, Brazil

## Abstract

**Background:**

A paradoxical immunologic response (PIR) to Highly Active Antiretroviral Therapy (HAART), defined as viral suppression without CD4 cell-count improvement, has been reported in the literature as 8 to 42%, around 15% in most instances. The present study aims to determine, in a cohort of HIV infected patients in Brazil, what factors were independently associated with such a discordant response to HAART.

**Methods:**

A case-control study (1:4) matched by gender was conducted among 934 HIV infected patients on HAART in Brazil. Cases: patients with PIR, defined as CD4 < 350 cells/mm^3 ^(hazard ratio for AIDS or death of at least 8.5) and undetectable HIV viral load on HAART for at least one year. Controls: similar to cases, but with CD4 counts ≥ 350 cells/mm^3^. Eligibility criteria were applied. Data were collected from medical records using a standardized form. Variables were introduced in a hierarchical logistic regression model if a p-value < 0.1 was determined in a bivariate analysis.

**Results:**

Among 934 patients, 39 cases and 160 controls were consecutively selected. Factors associated with PIR in the logistic regression model were: total time in use of HAART (OR 0.981; CI 95%: 0.96-0.99), nadir CD4-count (OR 0.985; CI 95%: 0.97-0.99), and time of undetectable HIV viral load (OR 0.969; CI 95%: 0.94-0.99).

**Conclusions:**

PIR seems to be related to a delay in the management of immunodeficient patients, as shown by its negative association with nadir CD4-count. Strategies should be implemented to avoid such a delay and improve the adherence to HAART as a way to implement concordant responses.

## Background

Highly active antiretroviral therapy (HAART) has been introduced in the therapeutic arsenal for HIV infection since the 90's. The efficacy of HAART turned HIV infection into a chronic condition which made it possible to considerably improve its survival rate over the last 20 years [1-3]. Such an improvement was in consequence of the virologic suppression that occurs in approximately 70% of patients during the first regimen of HAART [4], thus making their immunologic reconstitution possible [1,5].

The frequency of a paradoxical immunologic response to HAART, defined as viral suppression without CD4 cell-count improvement, has been reported in the literature as 8 to 42%, around 15% in most instances [6-25].

Several outcomes have been related to this poor immunologic response. Most of the studies had demonstrated worse outcomes when compared to those with complete response such as the increased risk of AIDS-defining illness or death [14,16,23,25]. Some authors, however, did not observe statistically significant differences between these outcomes in full responders and non-immunologic responders [26,27].

The efforts to determine the prevalence of this condition and its associated factors are hampered by the absence of standardization in its definitions. Different studies establish different boundaries in the CD4 cell counts or different time frames to cross such boundaries in the characterization of an adequate immune response, making it difficult to compare most of the results [6,8,10,14,16,22,28].

Different associated factors have also been pointed out by several authors, like older age [7,10,12,18,20,22,28-35], lower [19,20,23,24,32,34-37] or higher [10,18,21,28,38-40] baseline CD4 cell count, lower nadir CD4 count [20,22]; poor adherence to HAART [10,41]; regimen of HAART [10,31,32,38,42]; levels of baseline viral load [8,10,12,15,18,21-23,28,32,33,36] and even comorbidities [20,43] and HIV transmission category [6,8,28,34].

The present study aims to determine, in a cohort of HIV infected patients in Brazil, what factors were independently associated with such a discordant response to HAART.

## Methods

### Study Population and Design

Study individuals were selected from a cohort of 934 patients under treatment with HAART in a specialized facility in the city of Vitória, Espírito Santo State, Brazil. The study was designed as a matched case-control approach. Each case was matched to four controls on gender. Retrospective analysis was performed by a single investigator who collected the data from charts using a standardized form containing demographic, socioeconomic, and clinical and laboratory variables.

Cases were all patients with paradoxical immunologic response (PIR) being defined as a CD4 cell count below 350 cells/mm^3 ^in a patient with viral load suppressed who was on HAART over a year in the moment of the sampling. Controls were patients with the same characteristics as described for the cases, except for a CD4 count greater than or equal to 350 cells/mm^3^.

This level of CD4 was used because hazard ratios as high as 10.7 were found for AIDS or death when comparing individuals with counts below 200 cells/mm^3 ^with those with counts above 350 cells/mm^3^. Even comparison with individuals with counts between 200 and 350 cells/mm^3 ^revealed a hazard ratio as high as 8.5 [44]. Regarding viral suppression, the limit of detection most often used over the past years was 400 copies/ml, whereas in the study period (April-September 2009) the limit was 50 copies/ml.

The controls were consecutively selected from the total number of patients who met the matching criteria from cases. As cases and controls were defined by the level of CD4 cell count, such count should be consistently determined by a constant value measured at least for twice during the previous six months (April-September 2009). For the purpose of this study, previous year was that between Oct. 1, 2008 and Sept. 30, 2009.

Sample size was calculated assuming an 80% power for the test, a 95% confidence interval in the estimation of the effect, a minimum frequency among controls of 25% for any of the assessed risk factors, a ratio of one case to four controls and a frequency of the risk factors three times higher in the group of cases. With these parameters, 30 cases and 120 controls were necessary to discriminate the difference estimated a priori. This calculation was conducted using Epi Info™ Version 3.5.1.

This study was approved by local Institutional Committee of Ethics on research on Sept. 9, 2009 under the registration number 087/09. Data collection was performed from Oct. 1, 2009 to Jan. 31, 2010.

### Eligibility Criteria

Inclusion criteria were: age greater or equal to 18 years old; having started HAART according to Brazilian guidelines, with CD4 < 350 cell/mm^3^; use of HAART for more than one year at inclusion; at least two CD4 counts in the previous six months; HIV viral load < 400 copies/ml (most common limit of laboratory detection over time) during six months before inclusion, and to be registered at the local outpatient clinic.

Individuals were excluded if they: had participated in the programmed interruption of HAART at any time during their follow-up; underwent treatment with interferon or chemotherapy in the last year; were considered as immune failures in the previous year (defined as a decline of more than 25% of the CD4 count); were considered as clinical failures in the previous year (defined as the occurrence or recurrence of AIDS defining illness after 3 months of HAART); had irregular use of HAART in the previous year (defined as discontinuation of the regimen for more than 30 consecutive days); failed to attend the medical appointments in the previous 6 months, and were pregnant at the inclusion moment of the sampling process.

### Statistical analysis

Chi-square test or Fisher's exact test were used for comparison of categorical variables between the two groups. For continuous variables, "Student's" T test was used for comparison of normal distributions between the two groups. For non-normal distributions, it was used the Mann-Whitney test.

Multivariate analysis was performed by the binary logistic regression model using Enter method for all variables with a p-value < 0.1 in the bivariate analysis model. It is important to note that the inclusion of variables in this model was performed according to a hierarchical causal theoretical tree previously developed for this study. Such a hierarchy had previous application in epidemiological studies to improve the accuracy of statistical analysis [45].

The model was fitted with the selected variables that fulfilled the requirements for logistic regression: being independent, having p-value < 0.1 in bivariate analysis, having values in the cells of the cross tabs greater than or equal to one, and having not more than 20% of cells values below five. Variables were not included in the model if missing information exceeded 10% of the sample.

The independency among variables was tested to see if there was a correlation or association between them. Pearson correlation test or Chi-square test was performed, as more appropriate. When variables were associated or correlated with each other, the one chosen to enter the model was the most significant in the bivariate analysis or the most relevant in clinical practice.

The Hosmer-Lemeshow test was used to verify if the variables were well adapted to the theoretical model.

All statistical analyses were performed through the SPSS software (*SPSS *Statistics package version 15.0).

### Ethical Clearance

The study protocol was approved by the Ethics Comitee of the Center of Health Sciences of the Universidade Federal do Espírito Santo.

## Results

Out of 934 patients on HAART during the study period (April-September 2009) 563 had undetectable HIV viral load (HIVVL) (limit of detection: 50 copies/ml). Out of 563 with viral suppression, 39 cases and 160 controls were consecutively selected, with individual matching by gender. Controls were derived from 272 medical records sequentially verified. In the group of cases, there were eight females and 31 males, whereas in the control group 36 females and 124 males.

**Figure 1:**
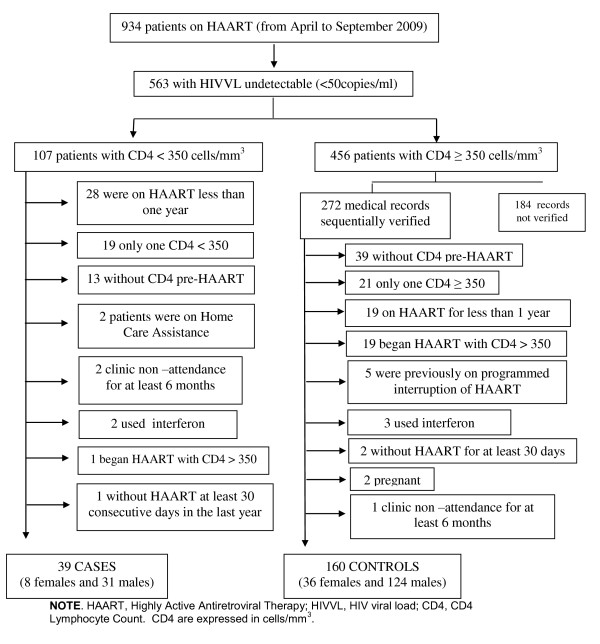
**Flow Chart of Eligibility Criteria**.

Sampling of cases and controls according to the eligibility criteria of the study is represented in Figure 1.

### Descriptive and bivariate analyses

Demographic and socioeconomic characteristics of the 199 patients (39 cases and 160 controls) included in the study are shown in Table 1.

**Table 1:** Demographic and socioeconomic characteristics among cases and controls.

Demographic and socioeconomic characteristics	Cases (n = 39^a^)	Controls (n = 160^a^)	Bivariate analysis
			p-value^c^
AGE AT AIDS DIAGNOSIS^b ^(years)	42.4 ± 14.3 Median: 38 (IQR: 32.7-49.5)	40,2 ± 10.9 Median: 38.50 (IQR: 31.5-48.7)	0.637
AGE AT HAART INITIATION^b^	42,8 ± 10 Median: 38 (IQR: 32.7-49.5)	40,54 ± 10.8 Median: 39 (IQR: 31.5-48.7)	0.630
RACE/SKIN COLOR - n (%)			0.440
White	14 (35.9)	75 (46.9)	
Mixed	17 (43.6)	55 (34.4)	
Black	6 (15.4)	22 (13.8)	
MARITAL STATUS - n (%)			0.989
Single	13 (33.3)	55 (34.4)	
Married	14 (35.9)	66 (41.2)	
Separate/divorced	3 (7.7)	17 (10.6)	
Widow	2 (5.1)	12 (7.5)	
OCCUPATION - n (%)			0,258
Unemployed	2 (5.1)	6 (3.7)	
Not specialized	8 (20.2)	52 (32.5)	
Autonomous	7 (18)	12 (7.5)	
Specialized	2 (5.1)	7 (4.3)	
Others	19 (25.6)	80 (50)	
EDUCATION - n (%)			0,656
Four years at most	6 (15.4)	33 (20.6)	
Until eight years	18 (46.1)	56 (35)	
High school	11 (28.2)	43 (30.6)	
Universitary education	2 (5.1)	14 (8.7)	

n, number; HAART, highly active antiretroviral therapy.

^a ^different values due to missing data.

^b ^Continuous data presented by mean ± standard deviation (SD) and by median with interquartile range (IQR).

**^c ^**Chi-square test or Fisher's exact test were used for comparison of categorical variables between the two groups, as more appropriate. For continuous variables, "Student's" T test was used for comparison of normal distributions between the two groups. For non-normal distributions, it was used the Mann-Whitney test.

The median age at diagnosis for AIDS and the onset of the first HAART regimen was 38 years old. From these subjects (cases plus controls) 37.3% were single, 44% married, 11% separated, and 7.7% widowed. Regarding race/color, there were 47.1% of white people, 38.1% mixed, and 14.8% black. The educational level of the patients was as follows: 19.7% had four years of education at most; 40.4% had up to eight years; 29.5% had completed high school, and 8.7% with University education (completed or not).

There was no statistically significant difference between groups for the following clinical data: HIV transmission category; Rio de Janeiro/Caracas or Centers of Diseases Control (CDC) adapted criteria for AIDS definition; comorbidities in the previous year; the year of HIV diagnosis; time from HIV diagnosis until the beginning of the first HAART (Table 2). AIDS diagnosis was based mainly on adapted CDC criteria (88.4% of reports), and 70% of AIDS cases reported had determination of less than 350 CD4 cells/mm^3^. Regarding the HIV transmission category, 97% of them had a suspected sexual exposure, 11.2% were injecting drug users, and only 0.5% was related to a blood transfusion (Table 2).

**Table 2:** Clinical characteristics among cases and controls.

Clinical characteristics	Cases (n = 39^a^) n (%)	Controls (n = 160^a^) n (%)	Bivariate analyses		
			p-value^e^	OR	CI 95%
HIV transmission by intravenous drug use			1.00	0.88	0.28-2.7
Yes	4 (10.2)	18 (11.5)			
No	35 (89.8)	139 (86.6)			
AIDS defining illness before HAART			0.04^b^	2.34	1.0-5.3
Yes	11 (28.2)	23 (14.4)			
No	28 (71.8)	137 (85.6)			
AIDS defined by Rio de Janeiro/Caracas criteria			0.53	1.38	0.49-3.85
Yes	5 (12.8)	27 (16.8)			
No	34 (87.2)	133 (83.1)			
AIDS defined by CDC adapted criteria			0.40	1.52	0.56-4.17
Yes	33 (84.6)	143 (89.3)			
No	6 (15.4)	17 (10.6)			
Comorbidities in the previous year			0.43	0.75	0.36-1.54
Yes	24 (61.5)	109 (68.1)			
No	15 (38.4)	51 (31.9)			
Irregular use of HAART in the previous year			0.07^c^	2.22	0.9-5.3
Yes	9 (23.1)	19 (11.9)			
No	30 (76.4)	141 (88.1)			
Concomitant medication in the previous year for more than 30 consecutive days			0.00^b^	0.29	0.1-0.6
Yes	30 (76.9)	79 (49.3)			
No	9 (23.1)	81 (50.7)			
Year of HIV diagnosis			0.32	1.99	0.64-6.11
Before 1996	5 (12.8)	11 (6.9)			
After 1996	34 (87.2)	149 (93.1)			
Year of AIDS diagnosis			0.09^c^	8.59	0.75-97.3
Before 1996	2 (5.1)	1 (0.6)			
After 1996	37 (94.8)	159 (99.4)			
Time from HIV diagnosis until first HAART (months)^d^	39.6 ± 52.2	12.8 ± 26.9	0.57		
	Median: 19.5 (IQR: 1.7-63)	Median: 5.5 (IQR: 2.2-10.7)			
Total time in use of HAART (months)^d^	36.1 ± 39,7	62.0 ± 32,5	0.00^b^		
	Median: 17 (IQR: 13.7-53.5)	Median: 60 (IQR: 33.2-96)			
Time in use of last HAART regimen (months)^d^	19.2 ± 18.3	44.2 ± 28.6	0.00^b^		
	Median: 15 (IQR: 12.2-15.2)	Median: 37 (IQR: 19.2-70.2)			

n, number; OR, odds ratio; CI 95%, confidence interval of 95%; HAART, highly active antiretroviral therapy; CDC, Centers for Disease Control and Prevention.

^a ^different totals are due to missing data.

^b ^p-value < 0,050.

^c ^p-value < 0,1; variables that could be included in multivariate analysis.

^d ^Continuous data are represented by mean ± standard deviation and by median with interquartile range (IQR).

^e ^Chi-square test or Fisher's exact test were used for comparison of categorical variables between the two groups, as more appropriate. For continuous variables, "Student's" T test was used for comparison of normal distributions between the two groups. For non-normal distributions, it was used the Mann-Whitney test.

Cases and controls differed in the presence or not of an AIDS defining illness before their first HAART regimen (p-value = 0.040); in the presence or not of an AIDS defining illness before any HAART (p = 0.04); if they had been using concomitant medication in the previous year for over thirty consecutive days (p = 0.002); in the total time in use of HAART in months (p = 0.001), and in time of use of current HAART in months (p < 0.001) (Table 2).

Regarding the components of the HAART regimen, cases and controls differed in the presence or not of Zidovudine (p = 0.021), and had a marginal difference in their frequency resulting from the association of nucleoside analogues reverse transcriptase inhibitors with non-nucleoside analogues (p = 0.099). There were no significant differences for the following variables: therapy containing tenofovir plus didanosine; regimen containing protease inhibitors; prior use of mono or dual antiretroviral therapy; occurrence of substitutions in the components of HAART since the first regimen in use; number of substitutions since the first HAART regimen in use; percentage of visits attended in the last year, and the percentage of absences in the pharmaceuticals dispensations in the last year (data not shown).

Differences were observed in the presence of comorbidities and in the frequency of concomitant medications as follows: presence of dyslipidemia in the previous year (p = 0.002, OR 0.226, 95% CI 0.083 to 0.613), use of fibrates (p = 0.006, OR 5.306, 95% IC 1.477 to 19.057), and prophylaxis with trimethoprim-sulfamethoxazole (p < 0.001, OR 0.026, 95% CI 0.007 to 0.103) (data not shown).

There were also differences in the reasons for HAART substitutions: prior abandonment or poor adherence (p = 0.018, OR 3.392, 95% CI 1.33 to 8.65), and Zidovudine myelotoxicity (p = 0.001, OR 23.3, 95% CI 2.64 to 206.59) (data not shown).

Regarding laboratorial data, the following are the different characteristics between cases and controls: lymphopenia before first HAART (p = 0.026) and before the current HAART (p = 0.007), CD4 count prior to the first HAART (p = 0.001) and before the current HAART (p < 0.001), nadir CD4 count (the lowest CD4-count ever presented by patient during outpatient follow-up) (p < 0.001), time of undetectable HIVVL (p < 0.001) and time of HIVVL below 1 000 copies/ml (p < 0.001). There were no statistically significant differences between the two groups regarding the HIV viral loads before the first HAART and before the current HAART, either in absolute numbers or in logarithm values (Table 3).

**Table 3:** Laboratorial characteristics among cases and controls.

	Cases (n = 39^a^)	Controls (n = 160^a^)	Bivariate analysis		
Laboratorial characteristics	n (%)	n (%)	p-value^c^	OR	CI 95%
Lymphopenia prior to first HAART			0.026^b^	2.6	1.0-6.5
Yes No	10 (25.6) 19 (48.7)	22 (13.7) 112 (70)			
Lymphopenia prior to current HAART			0.007^b^	3.4	1.3-8.8
Yes No	10 (25.6) 18 (46.1)	16 (10) 100 (62.5)			
CD4-count prior to the first HAART^d ^(cells/mm^3^)	119.2 ± 104.5 Median: 82.5 (IQR: 19-215)	189.1 ± 87.4 Median: 206 (IQR: 122.7-47.7)	0.001^b^		
HIVVL prior to first HAART^d ^(copies/ml)	96 251 ± 110 029 Median: 54 193 (IQR: 33 250-117 647)	263 711 ± 501 872 Median: 104 192 (IQR: 19 766-330 500)	0.294		
Logarithm of HIVVL prior to the use of the first HAART^d^	4.7 ± 0.4 Median: 4.7 (IQR: 4.5-5)	5 ± 0.7 Median: 5.1 (IQR: 4.3-5.5)	0.547		
CD4-count prior to the current HAART^d ^(cells/mm^3^)	125.2 ± 109.4 Median: 119 (IQR: 19-190)	288.2 ± 237.5 Median: 225 (IQR: 164-321)	0.000^b^		
HIVVL prior to the current HAART^d^ (copies/ml)	90 725 ± 114 158 Median: 54 192 (IQR: 9 360-117 647)	125 178 ± 168 825 Median: 47 800 (IQR: 16 413-205 773)	0.487		
Logarithm of HIVVL prior to the current HAART^d^	4.4 ± 0.9 Median: 4.7 (IQR: 3.9-5)	4.6 ± 1 Median: 4.8 (IQR: 4.2-5.4)	0.842		
Nadir CD4-count^d ^(cells/mm3)	85.3 ± 68.1 Median: 80.5 (IQR: 19-127.2)	177.8 ± 87.8 Median: 202 (IQR: 109.5-239.5)	0.000^b^		
Time of undetectable HIVVL^d ^(months)	9.2 ± 4.2 Median: 9.5 (IQR: 5-12.2)	28.8 ± 21.3 Median: 26.5 (IQR: 12.7-35.7)	0.000^b^		
Time of HIVVL below 1,000 copies/ml^d ^(months)	18.8 ± 20.3 Median: 12 (IQR: 8-20.2)	38.9 ± 25.2 Median: 28 (IQR: 22.2-65.2)	0.000^b^		

n, number; OR, odds ratio; CI 95%, confidence interval of 95%; HAART, highly active antiretroviral therapy; CD4, CD4 Lymphocyte Count; HIVVL, HIV viral load; Nadir CD4-count, the lowest CD4 ever presented by patient; Undetectable HIVVL, over time the most common limit of detection was less than 400 copies/ml.

^a ^different totals are due to missing data.

^b ^p-value < 0,050.

**^c ^**Chi-square test or Fisher's exact test were used for comparison of categorical variables between the two groups, as more appropriate. For continuous variables, "Student's" T test was used for comparison of normal distributions between the two groups. For non-normal distributions, it was used the Mann-Whitney test.

**^d ^**Continuous data are represented by mean ± standard deviation and by median with interquartile range (IQR).

### Logistic Regression Analysis

**Figure 2:**
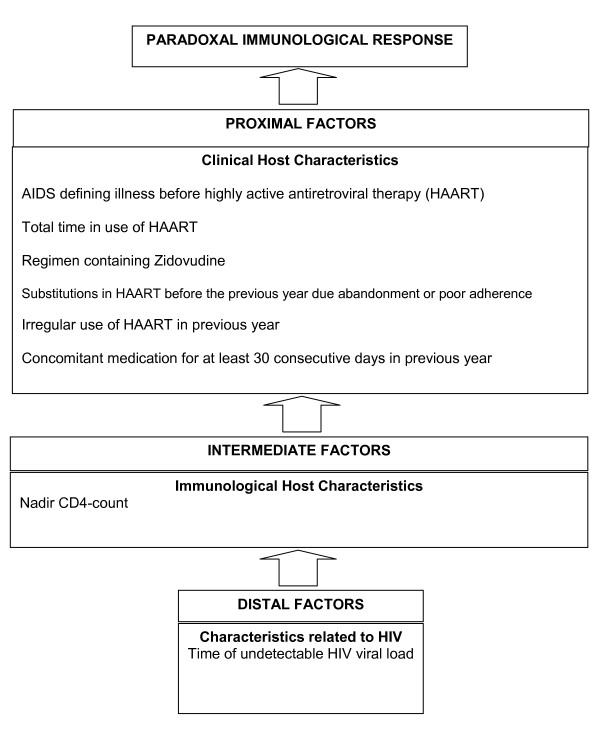
**Hierarchical causal theoretical model for data insertion in the multivariate analysis**.

Figure 2 presents the final hierarchical theoretical model used for multivariate analysis of the results.

Variables that remained significantly different after logistic regression were: total time in use of HAART (adjusted OR 0.981; CI 95%: 0.96-0.99), nadir CD4-count (adjusted OR 0.985; CI 95%: 0.97-0.99) and time of undetectable HIVVL (adjusted OR 0.969: CI 95%: 0.94-0.99) (Table 4).

**Table 4:** Logistic Regression Results.

Characteristics	Univariate analysis			Multivariate analysis^b^		
	p-value	OR	CI 95%	p-value	Aj OR	CI 95%
Total time in use of HAART	0.000^a^	-	-	0.013^a^	0.981	0.96-0.99
Concomitant medications over 30 consecutive days in previous year	0.002^a^			0.109		
Yes			-			-
No		0.29	0.13-0.65		0.423	0.14-1.21
Modifications in HAART before previous year due to abandonment or poor adherence	0.018^a^			0.159		
No			-			-
Yes		3.39	1.33-8.65		2.852	0.66-12.25
Regimen containing zidovudine	0.021^a^			0.359		
Yes			-			-
No		2.28	1.11-4.66		1.601	0.58-4.37
AIDS defining illness prior to HAART	0.040^a^			0.515		
Yes		0.88	0.28-2.77		1.475	0.45-4.74
No			-			-
Irregular use of HAART in previous year	0.071^a^			0.403		
Yes		2.22	0.91-5.39		1.888	0.42-8.36
No			-			-
Nadir CD4-count	0.000^a^	-	-	0.000^a^	0.985	0.97-0.99
Time of undetectable HIVVL	0.000^a^	-	-	0.037^a^	0.969	0.94-0.99

OR, odds ratio; CI 95%, confidence interval of 95%; Aj OR, adjusted odds ratio; HAART, highly active antiretroviral therapy; nadir CD4-count, the lowest CD4 ever presented by patient; HIVVL, HIV viral load; undetectable HIVVL, over time the most common limit of detection was less than 400 copies/ml.

^a ^p-value < 0,050.

**^b ^**Multivariate analysis was performed by the binary logistic regression model using Enter method for all variables with a p-value < 0.1 in the bivariate analysis model who fitted the requirements for logistic regression. It is important to note that the inclusion of variables in this model was performed according to a hierarchical causal theoretical tree previously developed for this study.

For total time in use of HAART, the odds ratio for each additional month was 0.981, i.e., for each additional month of ART use there is a reduction of 1.9% in the risk for paradoxical immune response (PIR). For each additional month of undetectable HIVVL there is a reduction of 3.1% in the risk of PIR (Table 4).

Regarding nadir CD4-count, every increase of one cell corresponded to a reduction of 1.5% in the risk of PIR (Table 4). Otherwise, each increase of 100 cells/mm^3 ^in nadir CD4-count corresponds to a risk reduction of PIR of 77.7%.

## Discussion

Paradoxical Immune Response (PIR) has been surrounded by uncertainties, both regarding its determinant factors and its consequences for the outcome of HIV infection. Despite its retrospective design and use of preexisting data, this study was able to uncover a negative association between PIR and total time in use of HAART as well as time for undetectable HIV viral load. Such an association was not adequately revealed by other studies because of their rigid eligibility criteria in terms of time frames, thus preventing any analysis of the influence of time frames on the outcomes [6,12,16,19-21,23].

Time of HIVVL suppression can be a surrogate marker of adherence to HAART. Thus, patients who have good adherence and, therefore, a longer time of undetectable HIVVL are less likely to come forward with paradoxical immune response. Such an association has also been reported by others [10,41]. Long time in use of HAART increases chances of optimal immune response. Several studies regarding this subject used predetermined goals to be met in terms of CD4-count. As an example, in the cohort study by Florence et al (2003) [20], it was considered adequate the immune response gain of ≥ 50 CD4-cells/mm3 or ≥ 75 cells/mm3 versus use of HAART for six (6) months or twelve (12) months, respectively. Dronda et al (2002) [6] considered adequate the immune response elevation of 100 cells/mm3 or higher at 24 months of HAART whereas for Kaufmann et al (2005) [23] the ideal immune response to be achieved after five years of ART was an elevation of 500 CD4 cells/mm3 or higher.

Another variable independently associated with PIR in this study was the nadir CD4-count. In other studies, both baseline CD4-count (measured before starting HAART) [10,18-21,23,24,28,32,34-40,46] and nadir CD4-count [20,22,37,47] have been associated with paradoxical immune response. As nadir CD4-count and baseline CD4-count are correlated, the present study used nadir CD4-count as an independent variable because it represents the lowest CD4 count value ever observed in a patient, along with his/her medical history, thus representing in fact the worst immune status he/she had ever had. The finding of an association between the increase in cell count at nadir CD4 determination and a decreasing risk of PIR conforms to Florence et al (2003) [20], Kaufmann et al (2002) [22] and Zoufaly et al (2010) [47] studies data.

Studies have been undertaken to elucidate the mechanisms involved in the occurrence of this poor immune response. Some of these studies have found direct association between the frequency of paradoxical immune response and the increase in T-cell activation (or persistence) and, consequently, excessive apoptosis of these cells [4,48,49]. Brenchley et al (2006) [50] observed that the bacterial translocation in these patients on HAART was the causation for the activation of their immune system.

Other studies have reported a relationship between PIR and cytokines profile. As an example, Sachdeva et al (2008) [36] demonstrated a low capacity to produce interferon alpha in patients with paradoxical immune response. Other authors have found associations with a reduced performance of interleukin 7 (IL-7) in the body, which was probably due to a down regulation of its receptor (CD127) during HIV infection [51]. Based on these conclusions, Levy et al (2009) [52] conducted a phase I/II clinical trial using IL-7 in patients with PIR, and demonstrated that this cytokine induced for a higher increase of CD4 cells when compared to that observed in the placebo group. A positive effect was also observed in the functional activity of CD4 cells in the group receiving IL-7 [52].

A relationship between age and PIR has been emphasized by studies highlighting a correlation with thymus size and function [7,10,12,18,21,22,28,29,31-34]. In the present study, age had not the importance evidenced by others.

Recent guidelines for the treatment of HIV infection do not establish a rigid CD4 cell count threshold at which therapy should be initiated. The International AIDS Society-USA Panel, for example, states that deciding when to initiate HAART is mainly a matter of the readiness of the patient [1]. Such recommendations are based on evidence that the benefits of treatment outweigh any additional risk posed by anti-retroviral drugs, as a long-standing untreated viremia implies a chronic inflammatory state resulting in end-organ damage and comorbid conditions. According to the literature, life expectancy in individuals with HIV infection is reduced even at higher CD4 cell counts [53]. Consequently, there is a concern to tailor the initiation of therapy according to other events related to HIV replication apart from CD4 cell-count limits, such as indications of rapid progression, older age and comorbid conditions [1]. Although the basic criteria in the present study are related to CD4 count as a determining factor for treatment, the results support the conclusion that early therapy could improve the immune response, as demonstrated by the inverse relationship between nadir CD4 count and the occurrence of PIR.

The limitations of the present study are those inherent to the case-control design, particularly regarding the vulnerability to selection bias and misclassification. Such possibilities are even more evident when one considers the preexistence of the data and the selection of prevalent cases. However, the consistence of the associations observed between the candidate variables of exposure and the outcome reinforces the validity of the conclusions, even if some potential risk factors could have persisted undetected.

## Conclusions

The main contribution of this study lies in the demonstration of an independent association of PIR with markers of intense immunossupression such as low nadir CD4 counts and short periods of time for undetectable viral loads. It seems that immune reconstitution is a process that depends on a residual immune function to be effective. In other words, if the immunossupression had gone too far, it could be too late to obtain a consistent reconstitution.

Therefore, there is a need for more studies, mainly with a prospective design to fully establish the consequences of PIR. If there is an important impact over morbidity and quality of life, as anticipated, the conclusions from studies like this one should strengthen the need for early intervention with HIV infected patients regarding HAART. The more we learn about it, the more we understand the complex nature of HIV infection, and better strategies are made available to allow for safe and effective intervention for the immune reconstitution. In the future, inadequate responses like PIR should be eliminated at the best scenario.

## Competing interests

The authors declare that they have no competing interests.

## Authors' contributions

JASC contributed to the study design, collection and analysis of data and writing of the manuscript. LNP participated in the study design and in the revision of the manuscript. FJPO made the analysis of data, organized the database and formatted the tables. CCJ participated in the study design, in the data analysis and in the revision of the manuscript. All authors read and approved the final manuscript.

## Pre-publication history

The pre-publication history for this paper can be accessed here:

http://www.biomedcentral.com/1471-2334/11/306/prepub
